# Stage‐Dependent β‐Synuclein Links MRI and Cognitive Decline in Alzheimer's Disease

**DOI:** 10.1002/acn3.70494

**Published:** 2026-07-20

**Authors:** Ulaş Ay, Merve Alaylioglu, Erdi Sahin, Sanem Sultan Yoruk Oner, Gulce Iskan, Gulce Cosku Yilmaz Cakan, Gozde Kizilates‐Evin, Emre Hari, Elif Kurt, Erdinc Dursun, Basar Bilgic, Hasmet Hanagasi, Hakan Gurvit, Tamer Demiralp, Duygu Gezen‐Ak, Bedia Samanci

**Affiliations:** ^1^ Neuroimaging Unit, Hulusi Behçet Life Sciences Research Laboratory Istanbul University Istanbul Turkey; ^2^ Department of Physiology, Istanbul Faculty of Medicine Istanbul University Istanbul Turkey; ^3^ Brain and Neurodegenerative Disorders Research Laboratories, Department of Neuroscience, Institute of Neurological Sciences Istanbul University‐Cerrahpasa Istanbul Turkey; ^4^ Behavioral Neurology and Movement Disorders Unit, Department of Neurology, Istanbul Faculty of Medicine Istanbul University Istanbul Turkey; ^5^ Department of Neuroscience, Aziz Sancar Institute of Experimental Medicine Istanbul University Istanbul Turkey

**Keywords:** Alzheimer's disease, beta synuclein, neurodegeneration, neuroimaging, synaptic biomarker

## Abstract

**Objective:**

Synaptic degeneration drives cognitive decline in Alzheimer's disease (AD), but synaptic biomarkers are scarce. Brain‐enriched β‐synuclein emerged as a synaptic damage marker. We investigated its diagnostic, prognostic, and structural correlates across the AD continuum.

**Methods:**

In a tertiary‐center cohort (*n* = 306), CSF β‐synuclein was measured. Cognitively unimpaired (CU), AD‐MCI, AD dementia (ADD), and non‐AD (FTD, PD, DLB, others) groups were included. ANCOVA compared groups (age/sex adjusted); ROC assessed diagnostic performance. Multivariable regression examined 2‐year MMSE decline associations. Voxel‐wise interaction models evaluated β‐synuclein–gray matter volume (GMV) relationships.

**Results:**

CSF β‐synuclein differed across groups (*p* < 0.001), with highest levels observed in AD‐MCI and lower levels in ADD. AD‐MCI levels exceeded CU/ADD. AD‐MCI versus CU AUC was 0.874. Baseline β‐synuclein predicted greater MMSE decline in AD‐MCI (*β* = 0.72, *p* < 0.001) and ADD (*β* = 0.47, *p* = 0.017). Voxel‐wise analyses revealed stage‐dependent β‐synuclein–GMV reversals in precentral and temporoparietal regions.

**Conclusion:**

CSF β‐synuclein shows a stage‐dependent pattern across the AD continuum, predicts cognitive decline, and dynamic structural coupling. It supports β‐synuclein as a relevant synaptic biomarker in AD.

## 1 Introduction

Alzheimer's disease (AD) is a progressive neurodegenerative disorder classically defined by the accumulation of amyloid‐β (Aβ) plaques and neurofibrillary tangles composed of hyperphosphorylated tau [1, 2, 3]. While these core pathologies are essential for diagnosis, synaptic loss and degeneration have been identified as the pathological hallmarks most strongly correlated with cognitive decline and disease severity [4, 5].

Synaptic failure occurs in the earliest stages of the disease, including preclinical AD and mild cognitive impairment (MCI), often preceding overt neuronal loss [6, 7, 8]. Understanding AD pathophysiology requires attention to mechanisms beyond the A/T/(N) system, including early synaptic dysfunction that may occur long before neuronal death [8, 9]. As disease‐modifying therapies emerge, there is a critical need for biomarkers [10, 11, 12] capable of detecting early synaptic injury, tracking disease progression, and monitoring treatment response beyond traditional amyloid and tau measures. Despite advances in amyloid and tau biomarkers, tools capturing active synaptic injury remain limited, and synaptic biomarkers may therefore represent a critical bridge between molecular pathology and clinical manifestation.

Beta (β)‐synuclein, a 14‐kDa presynaptic phosphoprotein and homolog of alpha‐synuclein, is abundantly and specifically expressed in the central nervous system, particularly within the thalamus, cerebellum, neocortex, hippocampus, and striatum [13, 14]. Unlike other synaptic markers that may be present in peripheral tissues, β‐synuclein is highly brain‐specific, making it a promising candidate for fluid‐based biomarker research [15, 16]. Previous studies have generally reported elevated β‐synuclein levels in the cerebrospinal fluid (CSF) and blood of AD patients, reflecting the release of synaptic components into the extracellular space following damage. Furthermore, emerging evidence suggests that β‐synuclein may serve as an early indicator of AD, with elevations detectable in the preclinical phase before significant neurodegeneration or cognitive impairment occurs [17, 18, 19, 20, 21, 22, 23, 24, 25].

While the utility of β‐synuclein in the AD continuum is increasingly recognized, its specificity across a broader spectrum of neurodegenerative diseases remains to be fully elucidated. Importantly, whether β‐synuclein reflects AD‐specific synaptic injury or a more general neurodegenerative process remains unclear. Recent research suggests that while β‐synuclein is markedly increased in AD and Creutzfeldt‐Jakob disease (CJD), it remains relatively stable in other conditions such as frontotemporal dementia (FTD), Parkinson's disease (PD), and amyotrophic lateral sclerosis (ALS) [15, 20, 26]. However, few studies have concurrently evaluated CSF β‐synuclein across AD, cognitively unimpaired individuals, and various non‐AD neurodegenerative syndromes within a single cohort. In addition to its potential as a fluid biomarker, emerging evidence suggests that β‐synuclein may be linked to structural brain changes. Oeckl et al. [26] demonstrated that increased levels of serum β‐synuclein significantly correlate with brain atrophy, particularly within temporal regions across the AD continuum, reflecting temporal‐specific synaptic degeneration.

This study aimed to investigate CSF β‐synuclein across the AD continuum and other neurodegenerative disorders in a large clinical cohort and to determine its diagnostic performance, relationship with canonical AD biomarkers, and prognostic value for cognitive decline and neurodegeneration. We examined group differences, evaluated associations with established biomarkers, assessed diagnostic performance for identifying AD at the MCI stage, and explored voxel‐wise relationships with regional gray matter atrophy using interaction‐based analytical approaches. We hypothesized that CSF β‐synuclein reflects synaptic injury, would be elevated along the AD continuum, distinguish AD from non‐AD neurodegenerative disorders, and associate with longitudinal cognitive decline and regional neurodegeneration. We further hypothesized that β‐synuclein provides complementary information beyond canonical biomarkers, reflecting synaptic dysfunction rather than core amyloid or tau pathology.

## 2 Methods

### 2.1 Participants

Participants were recruited from the Behavioral Neurology and Movement Disorders Unit, Department of Neurology, Istanbul Faculty of Medicine, Istanbul University, a tertiary referral center for cognitive disorders. The study was conducted in accordance with the Declaration of Helsinki and approved by the Institutional Review Board of Istanbul University Faculty of Medicine (27.01.2023–1600485). Written informed consent was obtained from all participants or their legal representatives. Caucasian patients born in Turkey were included.

Inclusion criteria comprised signed informed consent for CSF sampling and storage. Exclusion criteria included mixed dementia, major psychiatric or systemic illness. All participants underwent baseline Mini‐Mental State Examination (MMSE), Clinical Dementia Rating (CDR), and comprehensive neuropsychological assessment. A subset of AD patients had available 2‐year follow‐up MMSE, which was used to quantify longitudinal cognitive decline.

A total of 306 participants who underwent CSF examination for biomarkers were included: 27 cognitively unimpaired participants (CU), 24 CSF amyloid‐β negative (amyloid‐β1‐42 [−]) mild cognitive impairment (nAD‐MCI), 60 CSF amyloid‐β and pTau181 positive MCI (AD‐MCI), 73 AD dementia (ADD), 55 FTD (43 behavioral variant, 9 semantic variant primary progressive aphasia, and 3 nonfluent/agrammatic variant primary progressive aphasia), 47 PD, 10 dementia with Lewy bodies (DLB), and 10 other dementias, including corticobasal syndrome and progressive supranuclear palsy. CU subjects were recruited based on non‐specific cognitive complaints despite preserved independence in activities of daily living. Comprehensive neuropsychological testing, brain MRI, and CSF biomarker analyses were performed, all yielding normal results. They had MMSE score of 27–30 points at screening visit, and do not fulfill the criteria for mild or major neurocognitive disorder (MCI or dementia) according to DSM‐5 [27]. MCI patients had admitted to our clinic due to cognitive symptoms, but fully independent in daily life; had MMSE score of 24–30 points and a CDR of 0.5 (with a CDR sum of the boxes [SOB] score of ≤ 2); did not fulfill the criteria for any dementia (major neurocognitive disorder) according to DSM‐5. MCI patients were divided into two groups: those with CSF AD pathology (AD‐MCI, corresponding to Stage 3 AD according to the 2024 Alzheimer's Association Workgroup [AAW] criteria) and those without CSF AD pathology (nAD‐MCI) [3, 28]. Participants with ADD met the criteria defined by the Alzheimer's Association Workgroup (AAW stages 4–6) [3]. Other diagnoses were established according to current international consensus criteria [29, 30, 31, 32, 33, 34].

### 2.2 CSF Biomarkers and β‐Synuclein Analysis

All participants underwent lumbar puncture (LP) for the quantification of CSF biomarkers, including Aβ42, pTau181, total tau (tTau), and neurofilament light chain (NfL), in addition to peripheral blood sample collection. Following an overnight fast, LP was performed at 09:00 a.m. using a 25‐gauge needle. A total of 10 mL of CSF was collected into four polypropylene tubes and kept on ice for up to 9 h prior to centrifugation at 2000× *g* for 10 min at 4°C. The aforementioned biomarkers were analyzed on the same day. Subsequently, CSF samples were aliquoted into 1‐mL polypropylene tubes in 900‐μL volumes and stored at −80°C until further analysis.

Biomarker measurements were conducted at the Brain and Neurodegenerative Disorders Laboratory, Department of Neuroscience, Institute of Neurological Sciences, Istanbul University‐Cerrahpaşa, which participates in the University of Gothenburg quality control (QC) program. Quantification was performed using the INNOTEST β‐AMYLOID (1–42) ELISA kit (81576, Fujirebio), INNOTEST PHOSPHO‐TAU (181P) ELISA kit (81574, Fujirebio), INNOTEST hTAU Ag ELISA kit (81572, Fujirebio), and the NF‐light ELISA kit (10‐7001, UmanDiagnostics), respectively. All assays were carried out in accordance with the manufacturers' protocols, and each sample and standard was analyzed in duplicate.

CSF β‐synuclein levels were measured by using Human Synuclein‐beta ELISA kit (ELH‐SNCB‐1, RayBiotech; detection range: 24.6–5000 pg/mL; sensitivity: 24.6 pg/mL) according to the manufacturer's protocol. The samples and standards were run in duplicates. The inter‐run CV% was less than 10%.

CSF β‐synuclein was evaluated for its association with established CSF biomarkers and for differences across diagnostic groups. In the AD group, its relationship with clinical characteristics and 2‐year MMSE decline (reflecting cognitive deterioration) was also investigated.

### 2.3 Analytical Validation of the β‐Synuclein Assay

To assess the analytical performance of the RayBiotech Human Synuclein‐beta ELISA in human CSF, additional validation experiments were performed according to published immunoassay validation recommendations. Linearity was evaluated using serially diluted β‐synuclein‐spiked CSF samples. Parallelism was assessed in three CSF samples with high endogenous β‐synuclein concentrations using serial dilutions (undiluted, 1:2, and 1:4). Reproducibility was evaluated by repeat measurements of previously analyzed CSF aliquots. Recovery rates ranged from 79.1% to 105.1%, mean parallelism CV was 25.9%, intra‐assay CV was 11.0%, and inter‐assay CV was 14.1%.

### 2.4 MRI Acquisition and Processing

Structural MRI analyses were performed in participants for whom high‐quality T1‐weighted MRI scans were available and suitable for voxel‐based morphometry analyses (Supplementary Table 1). To ensure temporal correspondence between imaging and biomarker measurements, only participants who underwent MRI acquisition and CSF sampling during the same clinical evaluation were included. Inclusion in the MRI subgroup was therefore based solely on MRI availability and image quality criteria and was not influenced by clinical diagnosis, cognitive status, disease severity, or β‐synuclein levels. To evaluate the representativeness of the MRI subgroup, participants with and without MRI data were compared within each diagnostic group with respect to age, sex, MMSE, disease duration, and CSF β‐synuclein levels.

In a subset of participants with standardized MRI protocols (HC, *n* = 24; AD‐MCI, *n* = 23; ADD, *n* = 24; PD, *n* = 29; FTD, *n* = 17), high‐resolution structural T1‐weighted images were acquired using a 3‐Tesla clinical MRI system (Philips Achieva, Best, the Netherlands) equipped with a 32‐channel head coil at the Istanbul University Hulusi Behçet Life Sciences Research Laboratory Neuroimaging Unit. Anatomical images were obtained with a three‐dimensional T1‐weighted turbo field echo sequence aligned along the anterior–posterior commissural plane. Imaging parameters included TR = 8.1 ms, TE = 3.7 ms, flip angle = 7°, 176 contiguous slices, isotropic voxel size of 1 × 1 × 1 mm^3^, slice thickness = 1 mm, and a field of view of 256 mm. The total acquisition time was approximately 6 min.

Structural MRI preprocessing was performed using the Computational Anatomy Toolbox (CAT12.9, r2577) implemented in SPM12. Images underwent standard segmentation into gray matter (GM), white matter, and CSF, followed by spatial normalization to MNI space using DARTEL and modulation to preserve local volumetric information [35]. The resulting GM maps were smoothed with a 6‐mm full‐width at half‐maximum Gaussian kernel prior to statistical analysis.

### 2.5 Statistical Analysis

Statistical analyses were conducted using SPSS 28.0 and RStudio (v4.3.2). Continuous variables were summarized as mean ± SD or median (IQR). Group comparisons were performed using ANOVA or ANCOVA (adjusted for age and sex), with nonparametric equivalents when appropriate. Post hoc pairwise comparisons were corrected using the false discovery rate (FDR). Statistical significance was defined as *p* < 0.05 (two‐tailed).

Associations between CSF β‐synuclein and canonical AD biomarkers were examined using multiple linear regression models. Diagnostic performance was assessed using receiver operating characteristic (ROC) analyses. Adjusted ROC curves were derived from logistic regression models including β‐synuclein and relevant covariates (age and sex for AD‐MCI vs. CU; age, sex, and disease duration for AD‐MCI vs. non‐AD). AUC values were compared using the DeLong method, and optimal cut‐offs were determined using Youden's index.

Longitudinal associations between baseline CSF β‐synuclein levels and 2‐year MMSE change were evaluated using multiple linear regression models adjusted for age, sex, disease duration, and baseline MMSE. To formally assess whether β‐synuclein followed a non‐linear relationship with disease severity, we fitted a quadratic regression model with MMSE and MMSE^2^ as predictors, adjusted for age and sex. Longitudinal analyses were restricted to participants with available follow‐up MMSE assessments, and baseline characteristics did not differ significantly between participants with and without follow‐up data (Supplementary Table 2).

To investigate potential interactions between β‐synuclein levels and regional gray matter volume across diagnostic groups, a voxel‐wise full factorial design was implemented in CAT12. The model included five groups (CU, AD‐MCI, ADD, FTD, and PD) as between‐subject factors, with CSF β‐synuclein concentration entered as a continuous covariate of interest. Age, sex, and total intracranial volume (TIV) were included as nuisance covariates. Group‐by‐β‐synuclein interaction effects were assessed using the general linear model framework.

Statistical maps were evaluated using a voxel‐level threshold of *p* < 0.001 (uncorrected) combined with cluster‐level family‐wise error (FWE) correction at *p* < 0.05. When significant clusters were identified, post hoc region‐of‐interest (ROI) analyses were performed to characterize the directionality of the interaction effects.

For post hoc analyses, mean GMV values were extracted from significant clusters using SPM‐based ROI summarization procedures. Individual GMV values were then analyzed using linear regression models implemented in MATLAB, testing the interaction between diagnostic group and β‐synuclein while adjusting for age, sex, and TIV. Scatter plots with group‐specific regression lines were generated to visualize differential β‐synuclein–GMV relationships across disease stages.

## 3 Results

### 3.1 Demographic and Clinical Characteristics

The cohort comprised 306 participants (148 females, 158 males) with a mean age of 64.2 ± 9.78 years and mean MMSE of 22.8 ± 6.07. Demographic and clinical characteristics across diagnostic groups are summarized in Table 1. Diagnostic groups differed significantly in age and MMSE scores (*p* < 0.001), while sex distribution did not differ.

**TABLE 1:** Demographic and clinical features of the cohort.

Variable	CU *N* = 27	nAD‐MCI *N* = 24	AD‐MCI *N* = 60	ADD *N* = 73	FTD *N* = 55	PD *N* = 47	DLB *N* = 10	Other *N* = 10	*p*
Age (year), mean (SD)	56.3 (10.04)	65.8 (7.18)	69.8 (7.43)	63.3 (8.45)	61.8 (9.60)	64.1 (11.32)	71.9 (6.21)	60.3 (10.67)	< 0.001
Disease duration (year), mean (SD)	—	2.4 (2.36)	2.8 (1.91)	4.3 (3.54)	3.1 (1.86)	—	4.3 (4.5)	3.9 (2.93)	0.433
Female *n* (%)	17 (63%)	10 (42%)	32 (53%)	41 (56%)	23 (41%)	19 (40%)	3 (30%)	4 (40%)	0.4
MMSE, mean (SD)	29.4 (0.81)	27.9 (1.47)	26.7 (1.85)	17.7 (4.61)	21.0 (6.19)	29.0 (1.41)	24.0 (5.61)	17.2 (7.31)	< 0.001
CDR, range (median)	0 (0)	0–0.5 (0.5)	0.5 (0.5)	1–3 (2)	0.5–3 (1)	0–0.5 (0)	0.5–1 (1)	0.5–3 (1.5)	
MMSE difference at 2nd year, mean (SD)	—	—	2.1 (3.59)	7.0 (5.60)	—	—	—	—	< 0.001
β‐synuclein (pg/mL), mean (SD)	30.3 (9.75)	35.4 (8.06)	38.3 (5.59)	32.3 (9.64)	32.9 (9.18)	32.9 (6.12)	38.2 (5.27)	37.7 (8.07)	< 0.001

*Note:* Other includes corticobasal syndrome and progressive supranuclear palsy. FTD group included behavioral variant (*n* = 43), semantic variant primary progressive aphasia (*n* = 9), and nonfluent/agrammatic variant primary progressive aphasia (*n* = 3).

Abbreviations: AD‐MCI, mild cognitive impairment with CSF AD pathology; ADD, AD dementia; CDR, clinical dementia rating scale; CU, cognitively unimpaired; DLB, dementia with Lewy bodies; FTD, frontotemporal dementia; MMSE, mini‐mental state examination; nAD‐MCI, mild cognitive impairment without CSF AD pathology; PD, Parkinson's disease; SD, standard deviation.

### 3.2 CSF β‐Synuclein Across Diagnostic Groups

**FIGURE 1:**
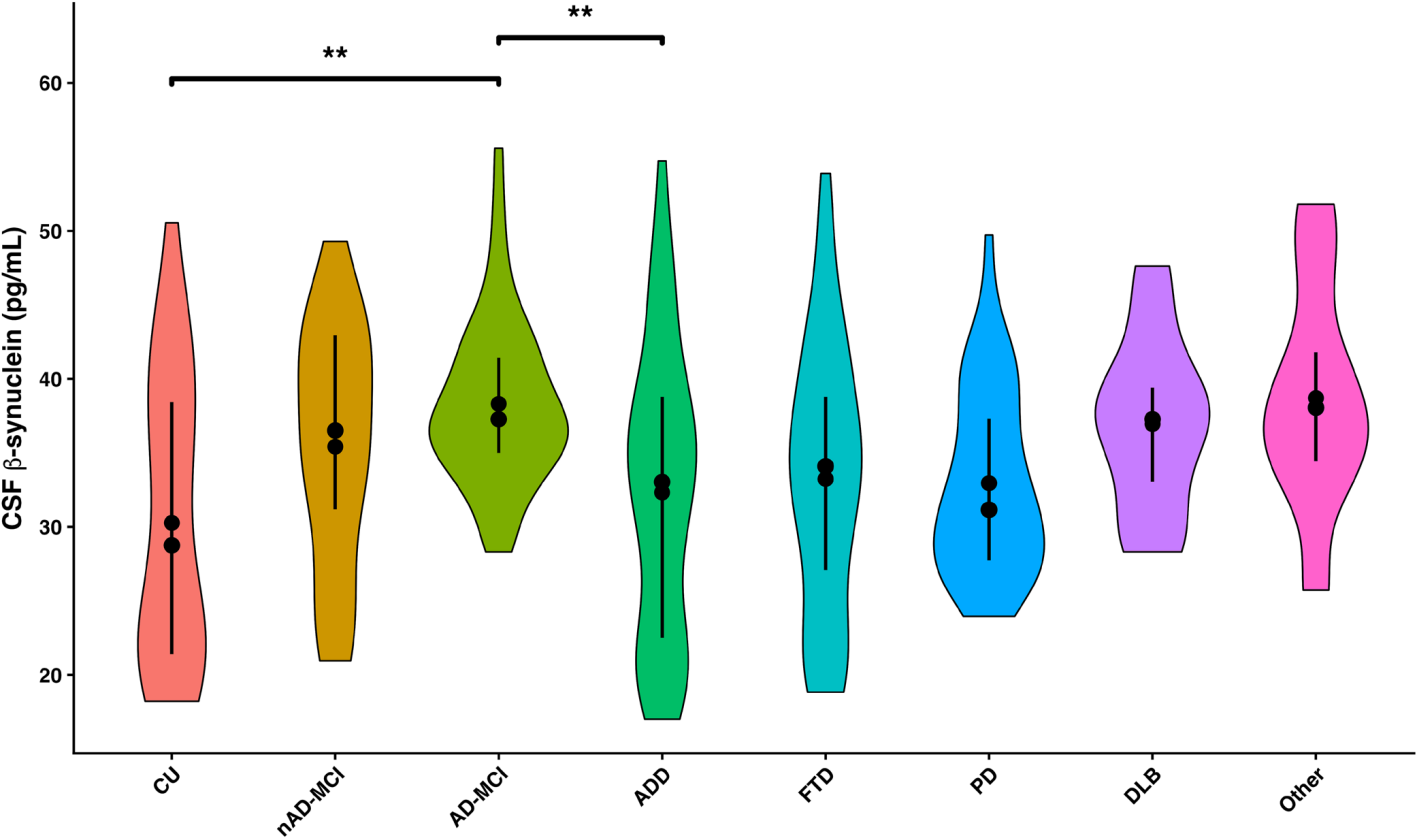
Cerebrospinal fluid (CSF) β‐synuclein levels across diagnostic. Violin plots depict the distribution of CSF β‐synuclein concentrations across diagnostic groups. The width of each violin represents the kernel density estimation of the data. Black dots indicate the group mean values, while the vertical black bars represent the interquartile range (25th–75th percentiles), with the central point marking the median. Group comparisons were performed using analysis of covariance (ANCOVA) adjusted for age and sex. Statistical significance between selected groups is indicated by brackets with asterisks (**p < 0.01). ADD, AD dementia; AD‐MCI, mild cognitive impairment with CSF AD pathology; CU, cognitively unimpaired; DLB, dementia with Lewy bodies; FTD, frontotemporal dementia; nAD‐MCI, mild cognitive impairment without CSF AD pathology; PD, Parkinson's disease. Other includes CBS (corticobasal syndrome), and PSP (progressive supranuclear palsy).

The mean β‐synuclein results were shown in Table 1. In an ANCOVA model adjusted for age and sex, CSF β‐synuclein levels differed significantly across diagnostic groups. Outliers in the AD‐MCI group (*n* = 3 values > 3 SD from group mean, identified via boxplot inspection) were excluded as potential assay artifacts or pre‐analytical variability. Sensitivity analyses excluding these outliers confirmed persistent group differences (*F*[7,294] = 3.55, *p* < 0.001, partial *η* ^2^ = 0.079), with unchanged post hoc findings. Post hoc FDR‐corrected analyses revealed significantly higher β‐synuclein levels in AD‐MCI compared with CU (*p*_FDR_ = 0.008) and ADD (*p*_FDR_ = 0.005). There was also a trend with PD (*p*_FDR_ = 0.061). No other pairwise comparisons remained significant after correction (Figure 1). β‐synuclein levels did not differ according to age at disease onset (< 65 vs. ≥ 65 years), either in the overall cohort or within the AD group.

### 3.3 Diagnostic Performance of CSF β‐Synuclein

**FIGURE 2:**
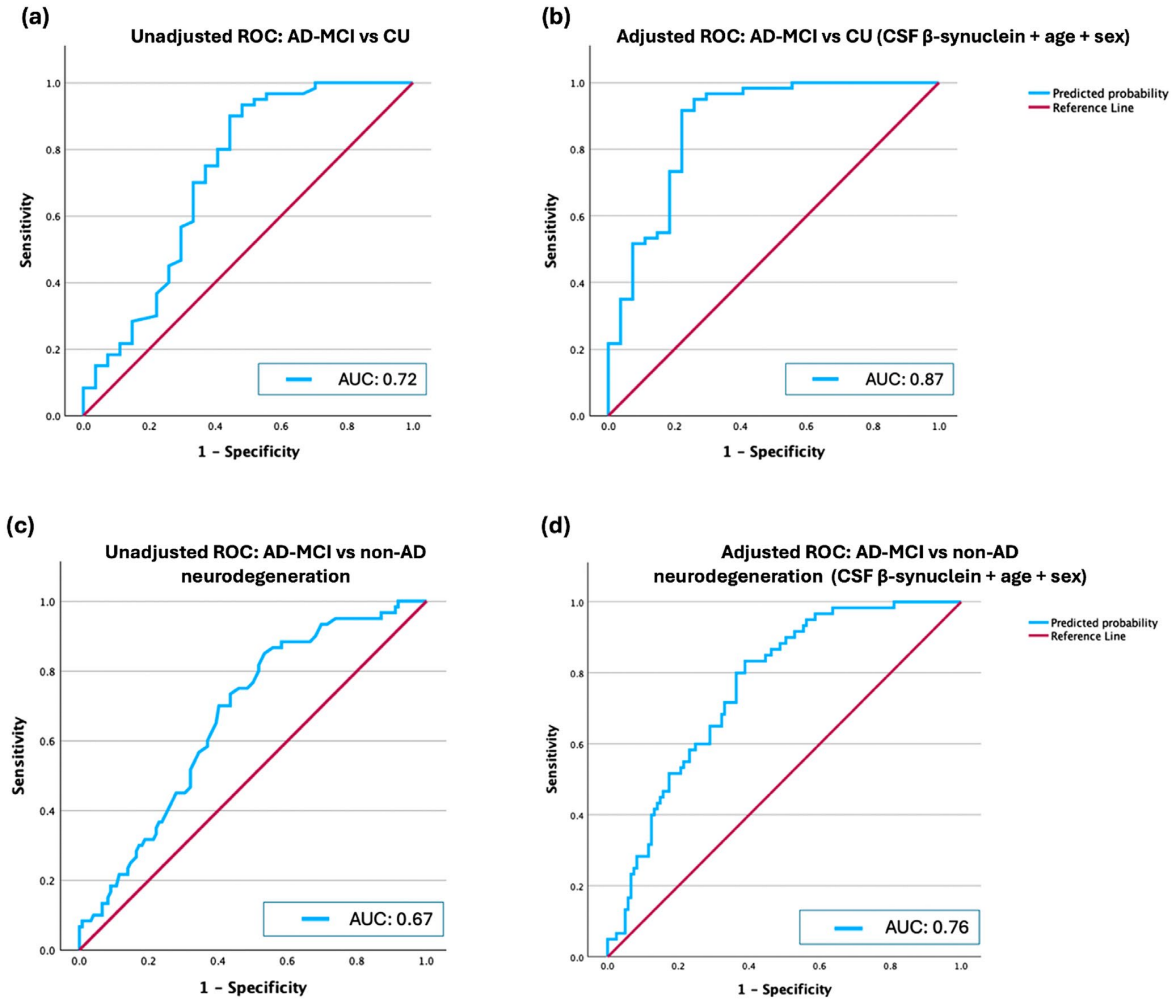
Receiver operating characteristic (ROC) curves for CSF β‐synuclein in differentiating MCI stage of Alzheimer's disease (AD‐MCI) from cognitively unimpaired (CU) participants (a, b), and non‐AD diagnoses except CU and MCI without AD pathology (c, d). Unadjusted models include β‐synuclein alone (a–c), whereas adjusted models include β‐synuclein, age at sampling, and sex (b–d). Values shown indicate area under the curve (AUC).

CSF β‐synuclein demonstrated moderate discriminatory ability between AD‐MCI (*n* = 60) and CU (*n* = 27) (AUC = 0.724, 95% CI 0.593–0.854). The optimal cutoff value for CSF β‐synuclein was 29.8 pg/mL, yielding a sensitivity of 90% and a specificity of 55.6% (Youden's index = 0.456). After adjustment for age and sex, discrimination improved substantially (AUC = 0.874, 95% CI 0.784–0.964). The optimal probability cutoff was 0.58, yielding a sensitivity of 91.7% and a specificity of 77.8% (Youden's index = 0.694) (Figure 2a,b).

CSF β‐synuclein demonstrated modest discriminatory ability between AD‐MCI and the non‐AD neurodegenerative group (FTD, PD, DLB, and other diagnoses; *n* = 122) in unadjusted ROC analysis (AUC = 0.669, 95% CI 0.589–0.748). The optimal cutoff value for CSF β‐synuclein was 32.62 pg/mL, yielding a sensitivity of 85% and a specificity of 46.7% (Youden's index = 0.317). After adjustment for age and sex, discrimination improved to a moderate level of accuracy (AUC = 0.762, 95% CI 0.693–0.831). The optimal probability cutoff was 0.164, corresponding to a sensitivity of 83.3% and a specificity of 61.2% (Youden's index = 0.445) (Figure 2c,d). Additional adjustment including disease duration did not materially change model performance, indicating that the discriminatory capacity of CSF β‐synuclein was largely independent of disease duration in this cohort.

### 3.4 Relationship With Canonical AD Biomarkers

In the overall cohort, multivariable regression adjusting for age and sex showed that lower CSF Aβ42 levels were significantly associated with higher β‐synuclein concentrations (*β* = −0.005, *p* = 0.003), whereas pTau and total tau were not independently associated. Age showed a modest positive association with β‐synuclein (*p* = 0.011). The overall model was significant (*F*[5,300] = 5.07, *p* < 0.001, *R* ^2^ = 0.081). In subgroup analysis, β‐synuclein was not significantly associated with canonical AD biomarkers even in AD‐MCI and ADD groups.

### 3.5 Longitudinal Cognitive Decline

There was no association between CSF β‐synuclein levels and baseline MMSE even in the overall cohort or diagnostic subgroups. However, higher baseline CSF β‐synuclein levels independently predicted greater 2‐year MMSE decline in AD‐MCI (*n* = 52) (standardized *β* = 0.72, *p* < 0.001; adjusted *R* ^2^ = 0.354) after adjustment for age, sex, disease duration, and baseline MMSE. A similar association was also observed in ADD (*n* = 59) (standardized *β* = 0.47, *p* = 0.017; adjusted *R* ^2^ = 0.160).

To formally assess the stage‐dependent pattern suggested by group comparisons, we fitted a quadratic regression model using MMSE as a continuous measure of disease severity, adjusted for age and sex. While MMSE was significantly associated with CSF β‐synuclein levels (*β* = 0.41, *p* = 0.017), the quadratic term was not significant (*β* = −0.005, *p* = 0.806), indicating that the observed group differences were not fully captured by a non‐linear MMSE‐based model.

### 3.6 MRI Analysis

No significant differences were observed between MRI and non‐MRI participants in the CU, AD‐MCI, ADD, or FTD groups with respect to age, sex, MMSE scores, or disease duration where applicable (Supplementary Table 1). CSF β‐synuclein levels did not differ between MRI and non‐MRI participants in the CU or AD‐MCI groups. However, lower CSF β‐synuclein levels were observed in MRI participants with ADD (21.9 ± 4.0 vs. 37.4 ± 7.2 pg/mL, *p* < 0.001) and FTD (24.8 ± 8.3 vs. 36.5 ± 7.1 pg/mL, *p* < 0.001). In the PD group, MRI participants were younger than non‐MRI participants (59.8 ± 11.7 vs. 71.1 ± 6.1 years, *p* < 0.001), whereas MMSE scores, sex distribution, and CSF β‐synuclein levels were comparable between groups.

### 3.7 Group × β‐Synuclein Interaction Effects on Gray Matter Volume

**FIGURE 3:**
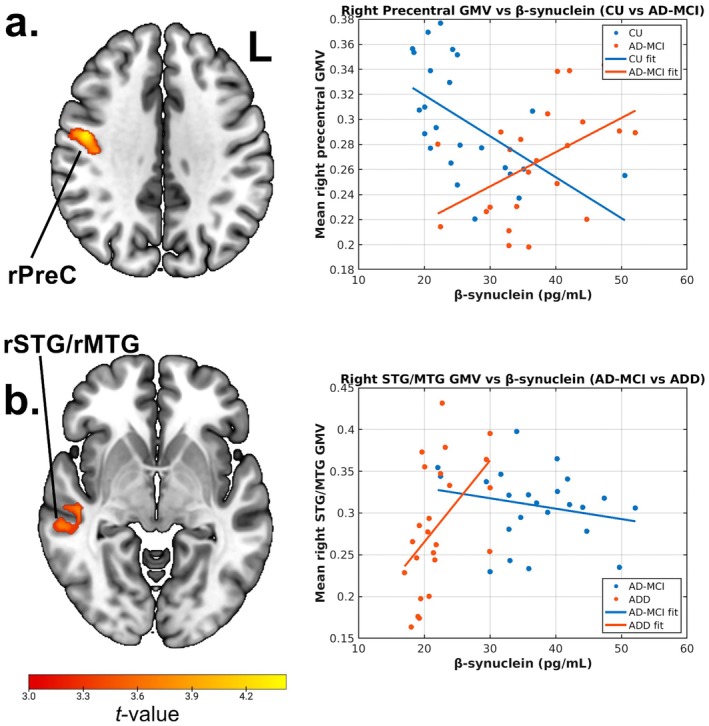
β‐synuclein–related gray matter interaction effects across Alzheimer's disease stages (a) Voxel‐wise interaction analysis identified a significant cluster in the right precentral gyrus (rPreC) for the AD‐MCI > CU contrast. The scatter plot illustrates post hoc ROI analysis showing opposite β‐synuclein–gray matter volume (GMV) associations between groups: CU exhibited a negative relationship between β‐synuclein levels and right precentral gray matter volume, whereas AD‐MCI participants demonstrated a positive slope, indicating a reversal of structure–biomarker coupling. (b) A second interaction cluster emerged at the right superior/middle temporal gyrus (rSTG/rMTG) junction in the ADD > AD‐MCI contrast. Post hoc ROI analysis revealed stage‐dependent modulation of β‐synuclein–GMV associations, with AD‐MCI showing a weak negative relationship and ADD exhibiting a more positive association. Clusters are displayed on the MNI template brain using a voxel‐level threshold of *p* < 0.001 (uncorrected) with cluster‐level FWE correction at *p* < 0.05. Scatter plots depict individual participants with group‐specific linear regression fits derived from ROI‐level interaction models adjusted for age, sex, and total intracranial volume. Color bars represent *T*‐values from the voxel‐wise interaction contrast. ADD, AD dementia; AD‐MCI, MCI with CSF Alzheimer's disease (AD) pathology; CU, cognitively unimpaired; L, left; rPreC, right precentral gyrus; rSTG/rMTG, right sıperior temporal gyrus/right middle temporal gyrus.

Voxel‐wise full factorial analyses revealed significant group × β‐synuclein interaction effects on regional gray matter volume (Figure 3). In the AD‐MCI>CU interaction contrast, a significant cluster was identified in the right precentral gyrus (cluster size = 751 voxels, peak *T* = 4.44, *p*_FWE_ = 0.008). In the ADD>AD‐MCI contrast, a second interaction cluster emerged at the junction of the right superior and middle temporal gyri (STG/MTG) (cluster size = 719 voxels, peak *T* = 4.22, *p*_FWE_ = 0.037).

#### 3.7.1 Post‐Hoc ROI Analyses

To further characterize the directionality of these interaction effects, post hoc ROI analyses were performed using mean gray matter volume extracted from significant clusters, while adjusting for age, sex, and total intracranial volume.

Post hoc analysis confirmed a significant group × β‐synuclein interaction within the right precentral gyrus (*β* = 0.0069, SE = 0.0015, *t* = 4.522, *p* = 5.35 × 10^−5^, 95% CI [0.0038, 0.0100]). Across participants, β‐synuclein levels showed a negative association with gray matter volume in the CU group (*β* = −0.0034, *p* = 0.0024), whereas AD‐MCI participants exhibited a comparatively positive slope, indicating opposite β‐synuclein–GMV relationships between groups. A significant main effect of group was also observed (*β* = −0.2494, *p* = 2.66 × 10^−5^), reflecting overall lower precentral GMV in AD‐MCI relative to controls. Among covariates, sex (*β* = −0.0396, *p* = 0.0038) and total intracranial volume (*β* = 0.000082, *p* = 0.0426) showed significant effects, whereas age was not associated with GMV (*p* = 0.77).

These findings indicate a reversal of the β‐synuclein–structure relationship in the early stage of AD, with higher β‐synuclein levels associated with lower cortical volume under normative conditions but relatively preserved or increased GMV in AD‐MCI.

Within the right STG/MTG cluster, ROI analysis demonstrated a significant group × β‐synuclein interaction (*β* = 0.0119, SE = 0.0032, *t* = 3.744, *p* = 5.71 × 10^−4^, 95% CI [0.0055, 0.0184]). In AD‐MCI participants, β‐synuclein showed a weak and non‐significant negative association with regional GMV (*β* = −0.0018, *p* = 0.24), whereas individuals with ADD displayed a significantly more positive relationship, indicating increased GMV with higher β‐synuclein levels relative to earlier disease stages. A significant main effect of group was present (*β* = −0.3052, *p* = 0.001), reflecting reduced temporal cortical volume in ADD compared to AD‐MCI. Sex remained a significant covariate (*β* = 0.0502, *p* = 0.0032), while age and TIV were not associated with GMV in this cluster (*p* > 0.90).

## 4 Discussion

In this study, we performed a comprehensive evaluation of CSF β‐synuclein across the clinical spectrum of AD and various non‐AD neurodegenerative syndromes. Our findings provide new insights into synaptic degeneration within current biomarker models. Specifically, we identified three primary takeaways: (1) a stage‐dependent pattern characterized by higher β‐synuclein levels in AD‐MCI than in both CU and ADD; (2) relative independence from core AD biomarkers within the AD group; and (3) a stage‐dependent reversal of the relationship between synaptic markers and GMV.

Our findings indicate that the most consistent and stage‐dependent changes of CSF β‐synuclein occur in the AD group. Consistent with previous reports [18, 19, 20], CSF levels were generally lower in non‐AD compared to the AD‐MCI. This pattern indicates that the most pronounced synaptic protein release occurs along the AD continuum. CSF β‐synuclein levels in AD‐MCI were significantly higher than in both the CU and ADD, indicating a distinct early‐stage peak rather than a progressive linear increase across the AD continuum [36]. While Barba et al. [15, 17] reported that β‐synuclein levels are elevated from the earliest clinical stages, our findings suggest that these levels do not continue to rise indefinitely. Although a formal quadratic regression model using MMSE as a continuous proxy of disease severity did not support a significant non‐linear association, group comparisons consistently demonstrated the highest β‐synuclein levels in AD‐MCI. This suggests that β‐synuclein may be more closely related to discrete disease stages than to cross‐sectional cognitive performance. Halbgebauer et al. [20] showed that AD‐MCI and ADD had significantly higher β‐synuclein levels than controls, with no significant difference between the two AD groups. One possible explanation for this pattern is a “synaptic exhaustion” phenomenon, whereby early disease stages are characterized by active synaptic injury and protein release, while advanced stages may show reduced β‐synuclein shedding because of progressive depletion of the available synaptic pool [20, 36, 37]. However, alternative explanations should also be considered. For example, changes in synaptic protein turnover, altered clearance mechanisms, selective vulnerability of β‐synuclein–enriched neuronal populations, or differences in disease stage composition may also contribute to the observed decline in β‐synuclein levels during the dementia stage. Therefore, the biological basis of this stage‐dependent pattern remains to be established in longitudinal studies. The lack of differences between AD and other neurodegenerative groups may be explained by demographic adjustment, disease heterogeneity, and the widespread distribution of β‐synuclein beyond AD‐vulnerable regions. While CSF β‐synuclein was numerically higher in AD‐MCI compared to FTD and PD, these differences were not statistically significant, with only a trend observed for PD (*p*_FDR_ = 0.061). The trend in PD aligns with previous literature showing that while β‐synuclein is markedly elevated in AD, it remains relatively stable or only slightly increased in Parkinson syndrome [20]. Regional enrichment of β‐synuclein in temporal–limbic structures may partly explain this pattern.

Although β‐synuclein levels in the DLB subgroup (38.2 pg/mL) were numerically comparable to those observed in AD‐MCI (38.3 pg/mL), the small number of DLB participants in our cohort precludes reliable interpretation of this finding. Therefore, these observations should be considered exploratory and hypothesis‐generating only. Previous studies have suggested that β‐synuclein may also be altered in DLB through mechanisms related to synaptic injury [15, 36, 38]; however, larger and biomarker‐characterized DLB cohorts will be required to clarify the disease‐specific behavior of β‐synuclein in Lewy body disorders. Collectively, these findings suggest that β‐synuclein reflects a broader spectrum of synaptic vulnerability across neurodegenerative diseases, with its most prominent elevations occurring during the early phases of the AD continuum. Finally, small sample sizes and variability within non‐AD subgroups such as DLB and “Other” likely reduced statistical power to detect differences after FDR correction.

Evidence from prion diseases provides important context for interpreting the stage‐dependent trajectory of β‐synuclein observed in our AD cohort. Several studies have demonstrated markedly elevated CSF and blood β‐synuclein levels in CJD, with concentrations substantially exceeding those reported in AD and most other neurodegenerative disorders [39, 40]. In CJD, β‐synuclein levels appear to rise abruptly at symptom onset and remain highly elevated throughout the symptomatic phase, paralleling the rapid and widespread synaptic destruction that characterizes prion disease. Notably, brain tissue β‐synuclein levels are reduced in CJD despite markedly increased biofluid concentrations, supporting the concept that β‐synuclein is released from degenerating synapses into extracellular compartments. These observations suggest that β‐synuclein primarily reflects the intensity of ongoing synaptic injury rather than disease‐specific pathology. Within this framework, the early peak observed in AD‐MCI may represent a phase of active synaptic degeneration and protein release, whereas the subsequent decline in ADD may reflect progressive depletion of the available synaptic pool. Thus, findings from prion diseases support the interpretation that β‐synuclein is a dynamic marker of synaptic injury whose biofluid concentrations depend not only on the extent of neurodegeneration but also on the temporal dynamics of synaptic loss [39, 40].

Our results reinforce the diagnostic and prognostic utility of CSF β‐synuclein. The marker demonstrated high accuracy in distinguishing AD‐MCI from CU, particularly after adjusting for age and sex (AUC = 0.874), similar to previous reports. Barba et al. [17] reported that CSF β‐synuclein identifies patients with AD pathology with high accuracy, citing an AUC of 0.91 for the total AD continuum and an even higher 0.97 for preclinical AD versus controls. Similarly, Halbgebauer et al. [20] found an AUC of 0.84 for distinguishing AD from controls using a standard ELISA. Bayoumy et al. [21] also reported AUCs between 0.71 to 0.80 for distinguishing AD patients from controls using region‐specific assays. Together, these studies position β‐synuclein as a sensitive marker of early clinical transition. Distinguishing early AD from other neurodegenerative syndromes remains a key clinical challenge. We also showed that CSF β‐synuclein effectively differentiated AD‐MCI from a pooled non‐AD neurodegenerative group including FTD, PD, and DLB (adjusted AUC = 0.762). Oeckl et al. [26] similarly reported that serum β‐synuclein could discriminate AD from behavioral variant FTD (AUC = 0.75). These results suggest that β‐synuclein may preferentially reflect AD‐related synaptic injury rather than generalized neurodegeneration. This level of accuracy supports further evaluation of β‐synuclein as a potential tool for clinical stratification.

The optimal CSF β‐synuclein cut‐off value identified in our study (29.8 pg/mL for distinguishing AD‐MCI from CU) differs from various values reported in the literature, likely reflecting the lack of a universal reference standard and variations in assay platforms and epitope specificity [21]. While some previous studies [17, 20] using the “Ulm‐style” ELISA reported significantly higher cut‐offs (> 313–540.4 pg/mL), our results align more closely with recent findings from Bayoumy et al. [21]. Their research demonstrated that absolute concentrations vary substantially depending on the targeted protein region. Similar lower concentrations observed with our RayBiotech ELISA kit may reflect conformational changes or interactions with proteolytic peptides that obscure specific epitopes. Despite differences in absolute thresholds, the clinical significance remains highly consistent across studies: β‐synuclein reflects early synaptic damage and peaks in early AD. Importantly, our results demonstrate that β‐synuclein can be reliably quantified using commercially available assays. However, compared with the extensively characterized in‐house ELISA developed by the Ulm group, the commercial RayBiotech assay used in the present study has undergone more limited analytical validation in human CSF. Accordingly, its analytical performance should be interpreted more cautiously, particularly with respect to recovery, parallelism, and potential matrix effects. Therefore, absolute β‐synuclein concentrations and direct comparisons across studies using different assay platforms should be interpreted with caution.

Another critical finding was the independence of β‐synuclein from core AD biomarkers within the AD group. While lower CSF Aβ42 levels were associated with higher β‐synuclein in the overall cohort, none of the core biomarkers independently predicted β‐synuclein levels within subgroups, including the AD‐MCI and ADD. Although some studies reported correlations with AD biomarkers [41], our finding indicates that β‐synuclein provides unique information about synaptic status that is not merely a proxy for amyloid or tau burden in AD. This positions β‐synuclein as a robust and complementary biomarker capable of tracking active synaptic injury independently of hallmark pathologies.

In our study, we observed that while baseline CSF β‐synuclein level showed no significant cross‐sectional association with baseline MMSE scores, it was a significant independent predictor of longitudinal 2‐year MMSE decline in both AD‐MCI and ADD stages. This lack of cross‐sectional association is consistent with previous studies reporting no relationship between β‐synuclein and baseline MMSE [20]. A recent serum‐based study has also suggested that β‐synuclein predicts longitudinal cognitive decline [16], and our results reinforce the biological interpretation that β‐synuclein is a dynamic marker of active synaptic injury rather than a static surrogate for current cognitive impairment. This suggests that synaptic protein release may capture ongoing pathology before overt clinical decline becomes apparent. This positions β‐synuclein as a valuable prognostic tool.

Neuroimaging results represent a distinct field contribution. Importantly, CSF β‐synuclein likely reflects dynamic synaptic shedding rather than cumulative structural loss, which may explain the observed stage‐dependent shifts in structure–biomarker coupling. Previous studies have linked elevated synaptic markers to temporal brain atrophy [26]. Our voxel‐wise interaction analysis revealed stage‐dependent changes in the structure–biomarker relationship. In the right precentral gyrus, the negative association observed in CU (higher β‐synuclein associated with lower GMV) was reversed in AD‐MCI patients, who showed a comparatively positive slope. While previous research in FTD has reported negative correlations in this region [26], the reversal identified in our study suggests that during the initial transition to clinical impairment, synaptic protein release dynamics may not follow simple linear atrophy models, potentially reflecting stage‐dependent release–reserve dynamics rather than a linear injury–atrophy relationship. Although β‐synuclein has been proposed to mediate apoptosis and potentially neuroprotective effects [14], current human CSF data do not directly support a compensatory upregulation mechanism in vivo. These findings should therefore be interpreted cautiously. Rather than indicating injury preserving volume, this pattern likely reflects differences in synaptic reserve across disease stages. This modulation was also evident at the junction of the STG/MTG, a critical hub where AD pathology typically originates and β‐synuclein expression is highly enriched [42, 43, 44]. Here, the relationship shifted from weakly negative in AD‐MCI to positive in ADD. Although Oeckl et al. linked elevated synaptic markers to progressive temporal atrophy through correlation models, our finding of a positive association in advanced stages points toward a possible “synaptic exhaustion” phenomenon [36]. As total synaptic density becomes severely depleted in later disease, a drop in CSF protein may occur, meaning that only individuals with relatively preserved synaptic sources continue to shed higher levels of β‐synuclein. These findings highlight that β‐synuclein–structure relationships must be interpreted within a disease‐stage context.

A major strength is the concurrent evaluation of CSF β‐synuclein across the AD continuum and multiple non‐AD syndromes within a single cohort, combined with voxel‐wise interaction analyses that capture stage‐dependent structural correlates. The inclusion of 2‐year follow‐up data enabled the validation of β‐synuclein's independent predictive value for future cognitive decline. There were also some limitations. While the overall cohort was large, some subgroups were relatively small, which may limit statistical power. Some diagnostic subgroups, particularly DLB and the heterogeneous “Other” category, were relatively small, limiting statistical power and preventing robust conclusions regarding disease‐specific β‐synuclein profiles. In addition, the study was conducted at a single tertiary referral center and included exclusively participants of Turkish ancestry. Therefore, the generalizability of these findings to other populations, healthcare settings, and ethnically diverse cohorts remains to be established. Replication in larger multicenter studies with broader demographic representation will be important to confirm the observed stage‐dependent β‐synuclein profile and its structural correlates. Diagnoses were based on clinical criteria and core CSF biomarkers rather than autopsy, which is the gold standard for defining primary vs. mixed pathologies. Biomarker analyses were primarily cross‐sectional, although follow‐up MMSE data partially mitigated this limitation. Longitudinal analyses were restricted to participants with available follow‐up MMSE assessments. Although baseline characteristics did not differ significantly between participants with and without follow‐up data, the possibility of attrition‐related bias cannot be completely excluded. The MRI findings should be interpreted in light of the relatively small neuroimaging sample, particularly within the AD‐MCI and ADD groups. Although significant associations were identified, the modest subgroup sizes may have limited statistical power and increased uncertainty regarding voxel‐wise effect size estimates. Consequently, these findings should be considered exploratory and require replication in larger independent cohorts. Although participants included in the MRI analyses were selected based on MRI availability and image quality rather than clinical characteristics, some differences in biomarker distributions were observed between MRI and non‐MRI subgroups. Importantly, demographic and clinical disease severity measures were largely comparable within the AD‐MCI and ADD groups. Therefore, the neuroimaging findings should be interpreted primarily within the MRI‐available cohort and warrant replication in larger imaging datasets.

The increasing availability of ultrasensitive plasma β‐synuclein assays further enhances the translational potential of this biomarker. Recent studies have demonstrated robust blood‐based β‐synuclein measurements and strong diagnostic performance in prion diseases, supporting the feasibility of assessing synaptic injury through minimally invasive sampling. Whether the stage‐dependent pattern observed in CSF across the AD continuum is similarly reflected in plasma remains unknown. Given the growing interest in blood‐based biomarkers for neurodegenerative diseases, future studies examining the relationship between CSF and plasma β‐synuclein across different stages of AD will be of particular interest.

In addition, although the commercial ELISA was analytically validated in CSF, one recovery value fell slightly below the conventional acceptance range and parallelism results should be interpreted cautiously. Residual matrix effects therefore cannot be completely excluded. Furthermore, compared with the extensively validated in‐house Ulm ELISA, the commercial assay has undergone more limited validation in human CSF, and absolute β‐synuclein concentrations and cross‐study comparisons should therefore be interpreted with caution.

In conclusion, our study underscores CSF β‐synuclein as a promising early biomarker of AD‐related synaptic dysfunction. We identified a stage‐dependent pattern with the highest β‐synuclein levels observed at the MCI stage followed by a decline in the dementia stage, possibly reflecting limits of the available synaptic pool. Furthermore, the independence of β‐synuclein from core AD pathologies and its ability to predict longitudinal cognitive decline support its role as a complementary biomarker. Our discovery of a stage‐dependent reversal in the structure–biomarker relationship suggests that synaptic protein release dynamics are more complex than previously understood. These findings support the use of β‐synuclein for early diagnosis, patient stratification, and potential use as a trial outcome marker. Recent advances in ultrasensitive blood‐based assays have demonstrated the feasibility of plasma β‐synuclein measurements in neurodegenerative diseases. Whether the stage‐dependent CSF profile observed in our study is mirrored in plasma remains an important question for future research, as successful translation to blood‐based testing could substantially enhance clinical applicability and accessibility.

## Author Contributions

**Ulaş Ay:** conception of the work, collecting and analysis of data, literature review, drafting of the manuscript, finalizing the manuscript; **Merve Alaylioglu:** collecting and analysis of data, literature review, drafting of the manuscript; **Erdi Sahin:** collecting of data, literature review, drafting of the manuscript; **Sanem Sultan Yoruk Oner:** collecting of data, literature review, drafting of the manuscript; **Gulce Iskan:** collecting and analysis of data, literature review, drafting of the manuscript; **Gulce Cosku Yilmaz Cakan:** collecting and analysis of data, literature review, drafting of the manuscript; **Gozde Kizilates‐Evin:** collecting of data, investigation and interpretation, literature review; **Emre Hari:** collecting of data, investigation and interpretation, literature review; **Elif Kurt:** collecting of data, investigation and interpretation, literature review; **Erdinc Dursun:** analysis of data, literature review, critical revision of the manuscript; **Basar Bilgic:** collecting of data, literature review, critical revision of the manuscript; **Hasmet Hanagasi:** collecting of data, literature review, critical revision of the manuscript; **Hakan Gurvit:** collecting of data, literature review, critical revision of the manuscript; **Tamer Demiralp:** collecting of data, literature review, critical revision of the manuscript; **Duygu Gezen‐Ak:** conception of the work, analysis of data, interpretation, literature review, critical revision of the manuscript; **Bedia Samanci:** conception of the work, collecting and analysis of data, literature review, interpretation, drafting and finalizing the manuscript.

## Funding

The authors have nothing to report.

## Ethics Statement

The study was conducted in accordance with the Declaration of Helsinki and approved by the Institutional Review Board of Istanbul University Faculty of Medicine (27.01.2023–1600485). Written informed consent was obtained from all participants.

## Conflicts of Interest

The authors declare no conflicts of interest.

## Supporting information


**Supplementary Table 1.** Baseline characteristics of participants with and without MRI within each diagnostic group.
**Supplementary Table 2.** Demographic and clinical features of the completers versus non‐completers.

## Data Availability

Data are available upon reasonable request.
