# Supplementary material for: Stage‐Dependent β‐Synuclein Links MRI and Cognitive Decline in Alzheimer's Disease
Source: Ann Clin Transl Neurol. 2026 Jul 20:10.1002/acn3.70494. Online ahead of print. doi: 10.1002/acn3.70494 (PMC13394880; doi:10.1002/acn3.70494)
Supplement: Supplementary file 1 — Supplementary Table 1. Baseline characteristics of participants with and without MRI within each diagnostic group. Supplementary Table 2. Demographic and clinical features of the completers versus non‐completers. [file ACN3-9999-0-s001.docx]

Supplementary Table 1. Baseline characteristics of participants with and without MRI within each diagnostic group

| **Group** | **Variable** | **MRI Available**  **N = 23** | **MRI Not Available**  **N = 37** | **p** |
| --- | --- | --- | --- | --- |
| CU | Age (year), mean (SD) | 61.3 (4.0) | 56.6 (9.5) | 0.177 |
|  | Sex, (F/M) | 2/1 | 15/9 | 0.914 |
|  | Baseline MMSE, mean (SD) | 28.7 (0.6) | 29.2 (1.0) | 0.248 |
|  | β-synuclein (pg/mL), mean (SD) | 37.3 (7.1) | 26.2 (7.6) | 0.097 |
| AD-MCI | Age (year), mean (SD) | 70.7 (6.2) | 68.3 (9.0) | 0.267 |
|  | Sex, (F/M) | 22/15 | 10/13 | 0.238 |
|  | Disease duration (year), mean (SD) | 2.45 (1.8) | 2.74 (1.7) | 0.557 |
|  | Baseline MMSE, mean (SD) | 27.0 (1.1) | 27.4 (1.3) | 0.339 |
|  | β-synuclein (pg/mL), mean (SD) | 37.8 (5.5) | 37.1 (7.7) | 0.723 |
| ADD | Age (year), mean (SD) | 63.7 (7.9) | 62.5 (9.5) | 0.593 |
|  | Sex, (F/M) | 26/23 | 15/9 | 0.238 |
|  | Disease duration (year), mean (SD) | 2.7 (1.9) | 2.96 (1.9) | 0.529 |
|  | Baseline MMSE, mean (SD) | 17.05 (4.5) | 19.0 (4.7) | 0.133 |
|  | β-synuclein (pg/mL), mean (SD) | 37.4 (7.2) | 21.9 (4.0) | <0.001 |
| FTD | Age (year), mean (SD) | 60.9 (9.61) | 62.9 (9.26) | 0.495 |
|  | Sex, (F/M) | 19/19 | 4/13 | 0.082 |
|  | Disease duration (year), mean (SD) | 3.1 (1.63) | 3.2 (2.24) | 0.824 |
|  | Baseline MMSE, mean (SD) | 19.8 (7.07) | 22.6 (4.23) | 0.114 |
|  | β-synuclein (pg/mL), mean (SD) | 36.5 (7.07) | 24.8 (8.27) | <0.001 |
| PD | Age (year), mean (SD) | 71.1 (6.11) | 59.8 (11.72) | <0.001 |
|  | Sex, (F/M) | 8/10 | 11/18 | 0.763 |
|  | Baseline MMSE, mean (SD) | 29.2 (0.79) | 28.9 (0.75) | 0.317 |
|  | β-synuclein (pg/mL), mean (SD) | 34.4 (6.39) | 31.4 (4.79) | 0.101 |

CU: Cognitively unimpaired; AD-MCI: Mild cognitive impairment with CSF AD pathology; ADD: AD dementia; FTD: Frontotemporal dementia; PD: Parkinson’s disease; SD: Standard deviation, F/M: female/male, MMSE: Mini-mental state examination.

Supplementary Table 2. Demographic and clinical features of the completers vs non-completers

| Variable | Completers  AD-MCI  N = 52 | Non-completers AD-MCI  N=8 | p-value | Completers  ADD  N = 59 | Non-completers ADD  N = 14 | p-value |
| --- | --- | --- | --- | --- | --- | --- |
| Age (year), mean (SD) | 71.8 (7.5) | 72.3 (8.6) | 0.884 | 66.1 (7.9) | 68.9 (8.0) | 0.249 |
| Disease duration (year), mean (SD) | 4.9 (2.7) | 3.9 (1.2) | 0.333 | 5.9 (3.2) | 7.3 (3.5) | 0.158 |
| Female n (%) | 27 (52%) | 5 (63%) | 0.712 | 32 (54%) | 10 (71%) | 0.368 |
| Baseline MMSE, mean (SD) | 26.7 (2.0) | 25.6 (1.5) | 0.149 | 17.8 (4.8) | 17 (3.6) | 0.840 |
| β-synuclein (pg/mL), mean (SD) | 44.4 (31.7) | 35.7 (6.5) | 0.445 | 33.2 (9.5) | 28.8 (9.9) | 0.123 |

AD-MCI: Mild cognitive impairment with CSF AD pathology; ADD: AD dementia; SD: Standard deviation, MMSE: Mini-mental state examination.
